# First Report of *Meloidogyne javanica* Infecting Strawberry (*Fragaria* × *ananassa*) in the United States

**DOI:** 10.2478/jofnem-2023-0034

**Published:** 2023-08-24

**Authors:** Clemen J. Oliveira, Gabrieli Riva, Janete A. Brito, Ruimim Xue, Johan A. Desaeger

**Affiliations:** Department of Entomology and Nematology, Gulf Coast Research and Education Center, University of Florida, Wimauma, FL, 33598, USA.; Florida Department of Agriculture and Consumer Services, DPI, Nematology Section, P.O. Box 147100, Gainesville, FL 32614-7100, USA.

**Keywords:** Root-knot nematodes, strawberry, *Meloidogyne javanica*, *Meloidogyne* spp, genomics

## Abstract

Strawberry (*Fragaria* × *ananassa*) is native to temperate regions. However, it has been produced in tropical areas, as a seasonal crop including in Florida, USA during the winter months. In March 2022, root galls resembling those induced by root-knot nematodes (*Meloidogyne* spp.) were observed in declining strawberry plants ‘Winterstar^TM^ FL 05-107’ growing in an organic-certified research site in Hillsborough County, Florida, USA. To our knowledge, *M. hapla* is the only root-knot species reported to infect strawberry in Florida. Preliminary molecular analyses, including newly synthesized DNA sequences (TW81/AB28 = OQ469833 - OQ469836; D2A/D3B= OQ473043 - OQ473047) using extracted nematode females from the strawberry roots, initially identified the RKN as *M. javanica*. Nematode species confirmation was further performed using the morphology of the female perineal patterns and isozyme analysis, mainly esterase (EST) and malate dehydrogenase (MDH), DNA sequencing, (NAD5-F/NAD5-R) and the SCAR primer set (Fjav/Rjav), species-specific for *M. javanica*. Isozyme analyses, EST= J3, which is specific for *M. javanica* and MDH=N1, as well as the morphology of female perineal patterns, agreed with data previously reported for *M. javanica*. A pathogenicity test on strawberry ‘Winterstar^TM^ FL 05-107’ transplants was performed using 10,000 eggs of the original *M. javanica* population, which induced galls on strawberry plants (Gall index, GI = 4.1) with egg masses clearly visible outside of the roots, producing an average of 1,344 eggs/gram of fresh root and 9,201 ± 4,206 eggs/root system. No galls or egg masses were observed on non-inoculated plants. Tomato ‘HM 1823’ was used as a control for the viability of the inoculum and showed numerous galls and egg masses (GI=5.0;).

The newly obtained DNA sequences using NAD5-F/NAD5-R (OQ474970 – OQ474972) were compared with other sequences available in the GenBank and were shown to be 100% identical to five *M. javanica* populations from Polk County, Florida, USA (OM418745 – OM418749) and the complete mitochondrion genome of *M. javanica* (NC026556). To our knowledge, this is the first report of *M. javanica* infecting strawberry in the United States.

Strawberry (*Fragaria* x *ananassa*) is an herbaceous plant belonging to the *Rosaceae* family, native to temperate areas of the Northern Hemisphere. The fruits are rich in vitamin C and can be consumed fresh, as well as used in desserts such as pastries and pie fillings. In the United States, strawberry performs and produces well over the winter months (November-April) generating a total revenue of $282 million (USDA-NASS, 2017; Whitaker, 2020). In Florida, strawberry is planted in October, and fruit is harvested from late November until April. For more information on nematode management and strawberry cultivation in Florida, refer to Oliveira (2022).

In March 2022, symptoms typical of root-knot nematode (RKN) infections, consisting of root galling, stunted plants, and chlorotic leaves, were observed in strawberry plants ‘Winterstar TM FL 05-107’ growing in an organic-certified research site at the Gulf Coast Research and Education Center (GCREC), University of Florida, Hillsborough County, Florida. USA. In Florida, only *M. hapla* has been reported to cause yield loss in strawberry. Another RKN species known to infect strawberry is *M. arenaria,* which was reported in Brazil (Desaeger 2019; Brum et al. 2019).

## Materials and Methods

Preliminary molecular analyses used females extracted from the strawberry roots. The TW81 (5′-GTT TCC GTA GGT GAA CCT GC-3′) and AB28 (5′-ATA TGC TTA AGT TCA GCG GGT-3′) primer set (Tanha Maafi et al., 2003) was used to amplify the rDNA internal transcribed spacer gene (ITS1-5.8SITS2). Also, the forward D2A (5′-ACA AGT ACC GTG AGG GAA AGT TG-3′) and the reverse D3B (5′-TCG GAA GGA ACC AGC TAC TA-3′) primers (Subbotin et al., 2006) were also used to amplify the D2-D3 expansion segments of 28S rRNA gene. The newly obtained sequences were assembled using Geneious Prime® (v.2019.2.1. Biomatters Ltd.) and deposited in the GenBank under the accession (TW81/AB28: OQ469833 - OQ469836; D2A/D3B: OQ473043 - OQ473047). Initially, identification revealed that this RKN population belongs to *M. javanica*.

Additional soil samples were collected from the infected area and used to multiply the RKN population on tomato ‘HM 1823’ (*Solanum lycopersicum*) in a greenhouse for further nematode species identification and confirmation. At the time of soil collection, zucchini ‘Desert Organic’ (*Cucurbita pepo*) was already planted as a double crop in the same beds where strawberries were grown previously. Double cropping with a spring vegetable is a common practice among strawberry growers in Florida. Isozyme analysis (EST; MDH) (Brito et al., 2008; 2021) from females (N=65) extracted from zucchini also identified the RKN as *M. javanica*.

**Figure 1:**
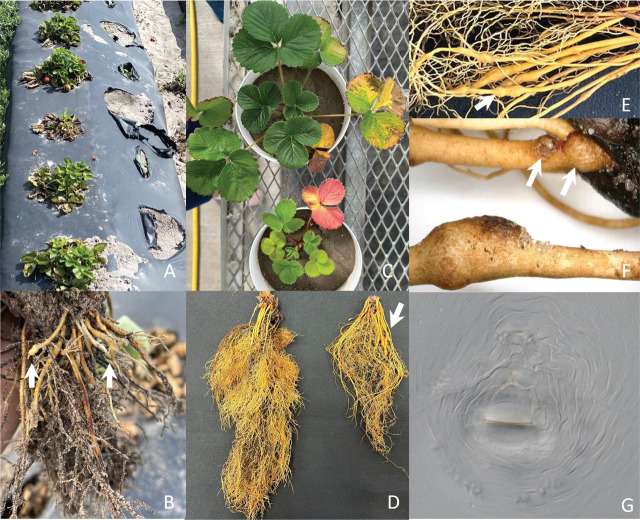
*Meloidogyne javanica* infecting strawberry (*Fragaria* × *ananassa* ‘Winterstar TM FL 05-107’). (A) Stunted plant growth observed in a naturally nematode-infested field; (B) Root system of a strawberry plant showing root galls; (C) Non-inoculated and inoculated (with *M. javanica*) plants in the greenhouse; (D) Root systems of uninoculated and inoculated plants; (E and F) Close-up of galls and egg masses (arrow) induced by the Javanese root-knot nematode under greenhouse conditions; (G) General morphology of the perineal pattern; notice the lateral lines.

For the pathogenicity test, Myakka fine sand (96.5% sand, 0.6% silt, and 2.9% clay) was steamed at 70°C for 12 hours using SST-15 120v Soil Sterilizer (Pro-Grow supply, Brooksville, WI, USA). This test was performed using ‘Winterstar TM FL 05-107’ transplants (N = 10) inoculated with 10,000 eggs/plant and a set of non-inoculated plants (N = 5). Those transplants were imported from a commercial nursery in California and planted in a 1.6 liters pot on October 22, 2022. Plants were watered twice a day and kept in a greenhouse. *Meloidogyne javanica-*induced galls (GI = 4.10) (Taylor and Sasser 1978) were observed on primary, secondary, and tertiary roots. The nematode egg masses were also clearly visible on the outside of the roots (Fig. 1), producing an average of 1,344 eggs/gram of fresh root and 9,201 ± 4,206 eggs/root system. Nematode eggs were extracted using 0.5% NaOCl, as described by Hussey and Barker (1973).

## Results

Galls induced by *M. javanica* seemed larger than those caused by *M. hapla,* which is a species commonly found on strawberries in Florida (Desaeger, 2019) However, further studies are necessary to elucidate this matter. Above-ground symptoms observed on inoculated plants included stunted plant growth and yellowing leaves similar to those observed in the field, with an average of 5.11 ± 1.10 and 9.94 ± 1.67 gram of fresh shoot weight per inoculated and non-inoculated plant, respectively, representing a 48.6% shoot weight reduction. Similarly, inoculated plants yielded an average of 7.1 ± 2.06 gram of fresh root weight, as compared to 9.48 gram ± 2.72 for non-inoculated plants. This represents a root weight reduction of 25%. No signs or symptoms of RKN infection were observed on the non-inoculated plants.

Tomato plants ‘HM 1823’ (N = 5), used as control for viability of the inoculum, showed abundant galls (GI = 5.0) and egg masses. Morphological analyses of the female perineal patterns (N = 9), isozyme (N = 22), mainly esterase (EST) and malate dehydrogenase (MDH), and molecular analyses, including DNA sequencing, were also performed using nematode specimens extracted from the strawberry roots. Morphology of the female perineal patterns were similar to those previously reported for *M. javanica* (Chitwood, 1949; Whitehead, 1968). Similarly, the species-specific phenotype EST = J3 for *M. javanica* and MDH = N1 were detected in all the females analyzed. This has been previously reported for this nematode species, which has been found infecting many plant species in Florida and other regions of the world (Brito et al., 2008; Cofcewicz et al., Pais and Abrantes, 1989).

Additionally, DNA was also extracted from single RKN females (N = ≥ 3) using NaOH (Hübschen et al., 2004), and amplification was performed with the NAD5-F (5′-TAT TTT TTG TTT GAG ATA TAT TAG-3′)/NAD5-R (5′-CGT GAA TCT TGA TTT TCC ATT TTT-3′) (Janssen et al., 2016) primer set, which targets the fragment of the mitochondrial gene NAD5 of the COII region, using thermocycle conditions by Janssen et al., 2016. Newly generated sequences (NAD5-F/NAD5-R: OQ474970 – OQ474972) were compared with those available in the GenBank using BLAST and showed 100% identity with other populations of *M. javanica* reported from Polk County, Florida USA (OM418745 – OM418749) and the complete mitochondrion genome of *M. javanica* (NC026556). The SCAR PCRs using species-specific primers Fjav/Rjav (Zijlstra et al., 2000) were used to confirm further the identity of the RKN found infecting strawberry. A fragment of 670 bp was obtained, which was expected for *M. javanica* (Zijlstra et al., 2000).

## Discussion

Considering that this nematode species is widespread in the state and commonly found infecting many crops and weed species in Florida, further studies are needed to determine the role of the strawberry cultivars in the infectivity of this nematode population and its effect on strawberry yield in Florida, as well as the phylogenetic relationship between this population found infecting ‘Winterstar TM FL 05-107’ and other populations of *M. javanica* found in Florida and different parts of the world. While no root galls were found on other strawberry cultivars that were in the same field as ‘Winterstar TM FL 05-107’, it would be useful to conduct more screening to evaluate if *M. javanica* can infect strawberry cultivars other than ‘Winterstar TM FL 05-107’. To our knowledge, this is the first report of *M. javanica* infecting strawberry in the United States.

## References

[j_jofnem-2023-0034_ref_001] Brito J. A., Perry R. N., Hunt D. J., Subbotin S. A. (2021). Isoelectric focusing of proteins. Techniques for work with plant and soil nematodes.

[j_jofnem-2023-0034_ref_002] Brito J. A., Kaur R., Cetintas R., Stanley J. D., Mendes M. L., McAvoy E. J., Powers T. O., Dickson D. W. (2008). Identification and isozyme characterization of *Meloidogyne* spp. infecting horticultural and agronomic crops and weed plants in Florida. Nematology.

[j_jofnem-2023-0034_ref_003] Brum D., Marchi P. M., Gonçalves M. A., Cruz F. F., Antunes L. E. C., Gomes C. B. (2019). Reaction of strawberry cultivars to root-knot and root-lesion nematodes. Horticultura Brasileira.

[j_jofnem-2023-0034_ref_004] Chitwood B. G. (1949). Root-knot nematod. Part 1. A revision of the genus *Meloidogyne* Goeldi, 1887. Proceedings of the Helminthological Society of Washington.

[j_jofnem-2023-0034_ref_005] Cofcewicz E. T., Carneiro R. M. D., Randing O., Chabrier C., Quénéhervé P. (2005). Diversity of *Meloidogyne* spp. on *Musa* in Martinique, Guadeloupe, and French Guiana. Journal of Nematology.

[j_jofnem-2023-0034_ref_006] Desaeger J. (2019). *Meloidogyne hapla*, the Northern Root-knot Nematode, in Florida Strawberries and Vegetables. *ENY070*.

[j_jofnem-2023-0034_ref_007] Floyd R., Abebe E., Papert A., Blaxter M. (2002). Molecular barcodes for soil nematode identification. Molecular Ecology.

[j_jofnem-2023-0034_ref_008] Hübschen J., Kling L., Ipach U., Zinkernagel V., Brown D., Neilson R. (2004). Development and validation of species-specific primers that provide a molecular diagnostic for virus-vector longidorid nematodes and related species in German viticulture. European Journal of Plant Pathology.

[j_jofnem-2023-0034_ref_009] Hussey R. S., Barker K. R. (1973). A comparison of methods collecting inocula of *Meloidogyne* spp. including a new technique. Plant Disease Reporter.

[j_jofnem-2023-0034_ref_010] Janssen T., Karssen G., Verhaeven M., Coyne D., Bert W. (2016). Mitochondrial coding genome analysis of tropical root-knot nematodes (*Meloidogyne*) supports haplotype based diagnostics and reveals evidence of recent reticulate evolution. Scientific Reports.

[j_jofnem-2023-0034_ref_011] Martin G. C. (1958). *Meloidogyne javanica* in strawberry roots.

[j_jofnem-2023-0034_ref_012] Martin G. C. (1961). Plants attacked by root-knot nematodes in the Federation of Rhodesia and Nyasalan. Supplementary list No. 2. Rhodesia Agricultural Journal.

[j_jofnem-2023-0034_ref_013] Pais C. A., Abrantes I. M. de. O. (1989). Esterase and malate dehydrogenase phenotypes in Portuguese populations of *Meloidogyne* species. Journal of Nematology.

[j_jofnem-2023-0034_ref_014] Oliveira C. J. (2022). Integrated Nematode Management in Florida Strawberry.

[j_jofnem-2023-0034_ref_015] Subbotin S. A., Sturhan D., Chizhov V. N., Vovlas N., Baldwin J. G. (2006). Phylogenetic analysis of Tylenchida Thorne, 1949 as inferred from D2 and D3 expansion fragments of the 28S rRNA gene sequences. Nematology.

[j_jofnem-2023-0034_ref_016] Tanha Maafi Z., Subbotin S. A., Moens M. (2003). Molecular identification of cyst-forming nematodes (Heteroderidae) from Iran and a phylogeny based on ITS-rDNA sequences. Nematology.

[j_jofnem-2023-0034_ref_017] Taylor A. L., Sasser J. N. (1978). Biology, identification and control of root-knot nematodes (*Meloidogyne* species).

[j_jofnem-2023-0034_ref_018] USDA-NASS (2017). https://www.nass.usda.gov/.

[j_jofnem-2023-0034_ref_019] Whitaker V. M., Boyd N. S., Peres N. A., Desaeger J., Lahiri S. (2020). Chapter 16. Strawberry Production. Vegetable Production Handbook for Florida, 2020–2021.

[j_jofnem-2023-0034_ref_020] Whitehead A. G. (1968). Taxonomy of *Meloidogyne* (Nematodea: Heteroderidae) with descriptions of four new species. Transactions of Zoological Society of London.

[j_jofnem-2023-0034_ref_021] Zijlstra C., Donkers-Venne D. T., Fargette M. (2000). Identification of *Meloidogyne incognita*, *M. javanica* and *M. arenaria* using sequence characterized amplified region (SCAR) based PCR assays. Nematology.

